# Bioconvection pattern of *Euglena* under periodical illumination

**DOI:** 10.3389/fcell.2023.1134002

**Published:** 2023-03-17

**Authors:** Nobuhiko J. Suematsu, Hiroshi Yamashita, Makoto Iima

**Affiliations:** ^1^ Graduate School of Advanced Mathematical Sciences, Meiji University, Tokyo, Japan; ^2^ Meiji Institute for Advanced Study of Mathematical Sciences (MIMS), Meiji University, Tokyo, Japan; ^3^ Graduate School of Integrated Life Sciences, Hiroshima University, Higashihiroshima, Japan

**Keywords:** bioconvection pattern, microorganisms, periodical environment, fluidic pattern, photosensitivity

## Abstract

Microorganisms respond to environmental conditions and often spontaneously form highly ordered convection patterns. This mechanism has been well-studied from the viewpoint of self-organization. However, environmental conditions in nature are usually dynamic. Naturally, biological systems respond to temporal changes in environmental condition. To elucidate the response mechanisms in such a dynamic environment, we observed the bioconvection pattern of *Euglena* under periodical changes in illumination. It is known that *Euglena* shows localized bioconvection patterns under constant homogeneous illumination from the bottom. Periodical changes in light intensity induced two different types of spatiotemporal patterns: alternation of formation and decomposition over a long period and complicated transition of pattern over a short period. Our observations suggest that pattern formation in a periodically changing environment is of fundamental importance to the behavior of biological systems.

## 1 Introduction

Microorganisms respond to environmental physicochemical conditions, such as chemical concentration (Colombetti and Diehn, 1978), gravity (Lebert and Häder, 1996), and light intensity (Diehn, 1969; Diehn, 1973; Creutz et al., 1978; Hader, 1987). Swimming microorganisms spontaneously generate macroscopic convection patterns originating from a response called “taxis” (Pedley et al., 1988; Hill and Pedley, 2005; Bees, 2020). When the environmental conditions prompt the organisms to swim upward, the cell density near the water surface increases and a top-heavy condition is generated. Once this unstable distribution is broken owing to fluctuation, the microorganisms settle locally and sink down owing to their own weight. Thereafter, upward swimming recommences, and the collective behavior of the population results in a spontaneous convection pattern. This phenomenon is known as “bioconvection.”

*Euglena* is a microorganism that responds to light intensity (Ogawa et al., 2016; Tsang et al., 2018; Noselli et al., 2019; Ozasa et al., 2019; Muku et al., 2023) and is known to form a localized bioconvection pattern under strong illumination from the bottom of the culture medium (Suematsu et al., 2011; Shoji et al., 2014). Usually, light strength is constant in time and homogeneous in space in experiments. However, in nature, the environmental conditions are neither constant nor homogeneous. Therefore, the response of bioconvection patterns to dynamic non-uniform illumination will yield a greater understanding of how microorganisms adapt to periodical changes in environmental conditions.

Here, we suggest temporally periodic illumination as the next simplest condition after constant uniform illumination. The bioconvection of *Euglena* was observed under spatially homogeneous illumination, whose light intensity changed periodically over time. With a long period rather than forming a pattern, bioconvection alternates formation and decomposition. By contrast, a complex spatiotemporal behavior was observed under a short period of flashing illumination. Based on our experimental observations, we suggest one of the response mechanisms of microorganisms to the periodically changing environmental conditions.

## 2 Experimental setup

*Euglena gracilis* was cultured in Koren–Hutner (KH) solution for 1 week (Koren and Hutner, 1967) and then inoculated into HYPONeX aqueous solution to observe bioconvection. The culture was diluted to the desired cell density and then placed in the dark for 1 h before use for bioconvection observation. The cell density was adjusted to 1.0 × 10^6^ cells/mL. All incubation processes were performed under 28°C and light conditions with 14 h bright and 10 h dark.

**FIGURE 1:**
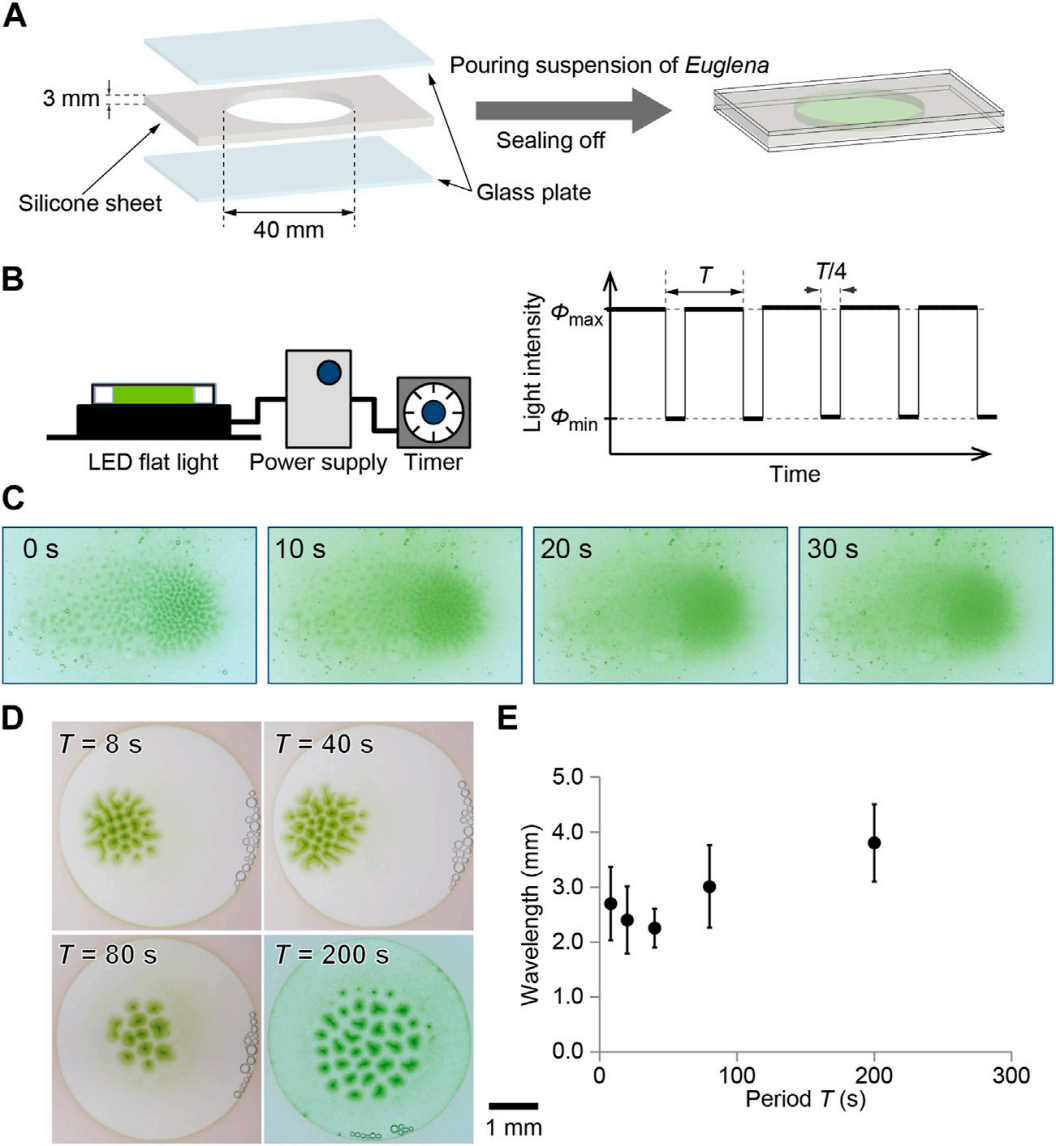
Illustration for **(A)** sample preparation and **(B)** temporal change in light illumination. A culture of *Euglena* was encapsulated using glass plates, where a silicone sheet was used as a spacer. The sample was illuminated from the bottom using flat light whose intensity was controlled with the *Arduino*. The light intensity was periodically changed between 0 and 3000 lx, where three quarters was bright and one quarter was dark. **(C)** Decomposition process of bioconvection pattern after stopping the illumination from the bottom. **(D)** Bioconvection pattern generated with temporal change in light intensity. The time periods were 8, 20, 40, 80, and 200 s. The snapshots obtained at 60 min are shown here for each period. **(E)** Characteristic wavelength for each pattern depending on the period. The snapshots obtained at 60 min were analyzed for each period.

The HYPONeX culture was soaked in a custom-made container in which a silicone sheet with a circular hole was placed on a glass plate and covered with another glass plate (Figure 1A). As a result, the culture solution was shielded into the hole of the silicone sheet with a thickness of 3 mm and diameter of 40 mm.

A bioconvection pattern was observed under temporally changing illumination intensity. A flat-light panel (LED Flat Light, Aitec System Co., Ltd., Japan) was connected to a controller (Arduino UNO). The light intensity changed between 0 lx (*ϕ*_min_) and 3000 lx (*ϕ*_max_) with periods (*T*) of 8, 20, 40, 80, and 200 s, where the period of bright was 3*T*/4 and of dark was *T*/4 (Figure 1B). The experiments curried out under room temperature, which was 24°C. Snapshots were captured using a digital camera (Eos Kiss X8, Canon, Japan). Images were analyzed using ImageJ (NIH, United States) on PC.

## 3 Results

### 3.1 Relaxation time for pattern formation and decomposition

The initial homogeneous distribution of cells became unstable under homogeneous illumination from the bottom, and then a convection pattern, which looked like a collection of spots, was formed approximately 300 s after beginning the illumination. The spots were generated almost entirely in the space at the initial stage and shifted slightly toward the center of the container. Finally, the spots gathered and formed a localized pattern (Suematsu et al., 2011). Once the formation of the localized pattern was complete, it was stable and was maintained for more than 1 h as long as the illumination was maintained.

To maintain this pattern, illumination from the bottom is necessary. If the light stops, the pattern is quickly dispersed within 30 s (Figure 1C). The time scale was 30 s, such that the pattern decomposition was faster than the formation process.

### 3.2 Temporal change illumination

With illumination, the homogeneous cell density distribution in the container started to fluctuate, and spot-shaped dense green regions gradually formed. After 60 min, the pattern approached a stationary state (Figure 1D). Even though the total amount of light illuminated to *Euglena* was the same, the stationary pattern depended strongly on the period of temporal illumination (Figure 1D). To estimate the characteristic wavelength of the localized pattern, neighboring spots were defined using Voronoi analysis and the distances between neighboring spots were measured. As a result, the average of the distances decreased with the period up to 40 s and then increased for more than 80 s (Figure 1E).

**FIGURE 2:**
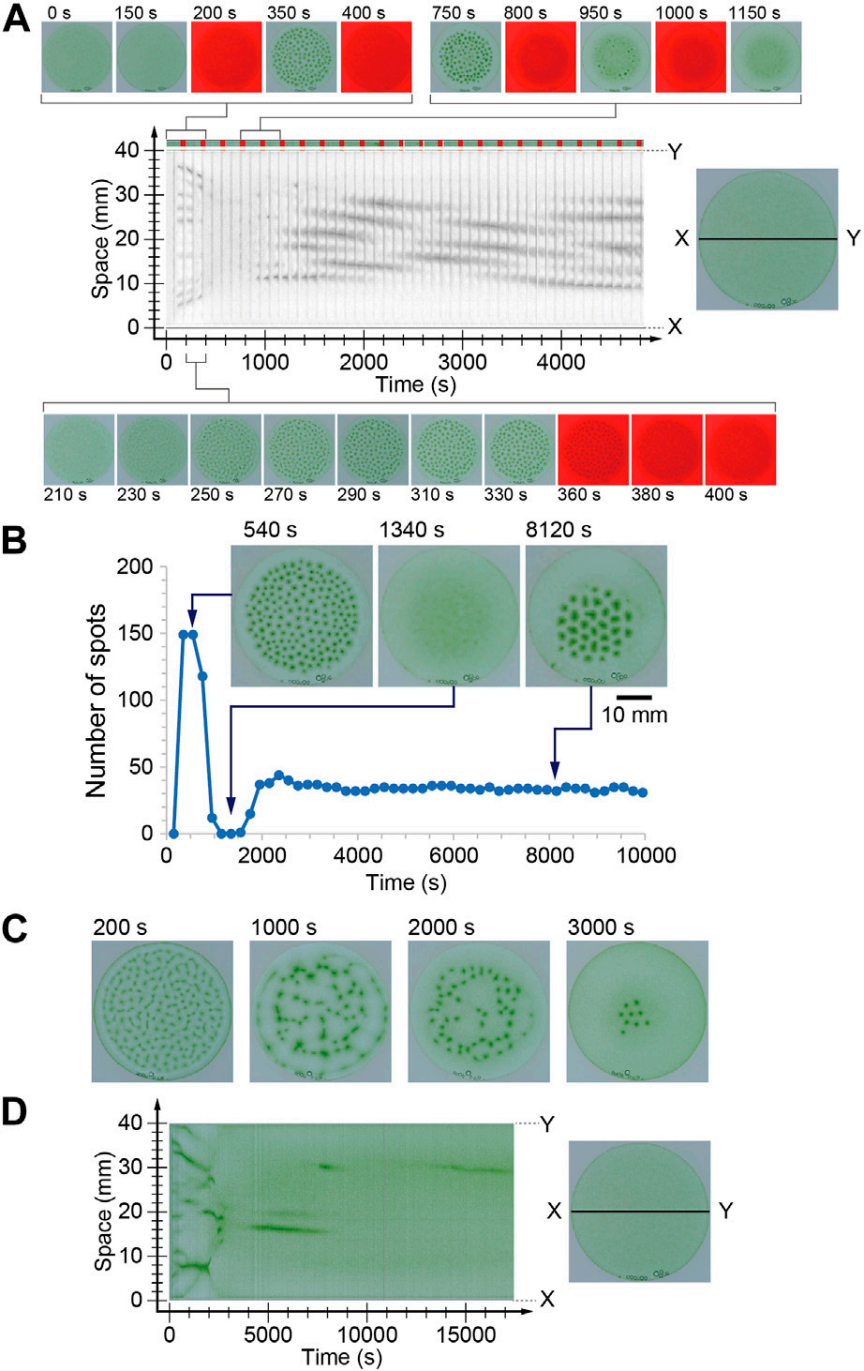
**(A, B)** Time series of the number of spots under the periodical illumination (200 s in the period). The space-time diagram in **(A)** was indicated gray scale image of red color. The dark periods are indicated in red on the color bar at the top of space-time diagram. **(C, D)** Time evolution of the bioconvection pattern under periodical changing light intensity whose period was 20 s.

In the case of a long period of 200 s, alternation between the pattern formation and decomposition processes was clearly observed during the initial three cycles. However, the next five cycles failed to form an ordered spot pattern. In these cycles, cells kept gathering, but the convection pattern was not completely formed. Thus, the spots or faint gathering spots collapsed during the dark period. However, the gathering at the center of the container progressed slightly, and convection pattern formation and decomposition were observed again (Figure 2A). This alternation was observed for more than 40 cycles and the spatial wavelength of the pattern was almost constant. At this time, decomposition was also observed during the dark period, but the pattern did not completely disappear. As a result, a stable pattern change in the oscillation was achieved (Supplemental Movie S1). To demonstrate this process, the number of spots was counted just before turning off the illumination for each period. The number suddenly increased in the initial two and three cycles and drastically decreased to zero in the next three cycles. Subsequently, the number increased again and approached 35 (Figure 2B).

The characteristic behavior was observed during transients of the pattern under temporal change illumination for 20 s in the period (Supplemental Movie S2). Under constant light, spots were generated at random positions and gathered monotonically. In the case of 20 s periodic light, the initial spot generation occurred similar to that observed under constant illumination (Figure 2C, 200 s). However, the spots were arranged in a network structure, which was very different from the localized pattern observed under constant illumination (Figure 2C, 1,000 s). Furthermore, the spots shifted toward random directions (Figure 2C, 2000 s), almost all spots disappeared, and several spots only survived locally (Figure 2C, 3000 s). At this time, the brightness of convection spots was a little brighter than that observed in the spots under constant illumination. Furthermore, the number of spots was nine, which was roughly one-fifth that of constant illumination. These results indicate that several cells swam in the region without convection. This was also quite different from the commonly observed convection pattern of *Euglena*. Usually, approximately all the cells gather in the convection region, and there are fewer cells in the region without convection.

## 4 Discussion

The convection pattern originated from the negative phototaxis of *Euglena*, which induced upward swimming of cells. The gathering of cells at the top of the culture lead to a density instability. Consequently, downward flows occurred at these times. Therefore, the downward flow appeared as a green spot when viewed from above. Once the illumination from the bottom stopped, the driving force was lost, and the pattern disappeared during a few tens of seconds. The wavelength of bioconvection pattern once decreased and then increased with increasing the period of the light intensity oscillation (Figure 1E). In addition, the temporal evolution of the pattern strongly depended on the period of external oscillatory illumination (Figure 2).

The swimming speed of the cells was approximately 100 μm/s and the depth of the container was 3 mm. Therefore, more than 30 s was required to reach the top. However, cells often change their direction. Therefore, it might take several hundred seconds to gather the cells at the top and induce convection. The period of 200 s of external light was comparable to the period of upward swimming of the cell. Based on this speculation, the disappearance of the spots in the initial stage (Figure 2A) was induced due to the lack of a driving force for the long period case.

In the initial two or three cycles, the cells were sensitive to illumination because they were cultured in the dark before starting the experiments. Therefore, convection spots were generated in a short time. However, illumination for several hundred seconds induced adaptation of the cells; thus, the sensitivity was slightly weakened. Owing to the weakness of the light sensitivity, the gathering at the top also slowed. As a result, the cells might fail to fulfill the convection-inducing condition for 150 s illumination. Accordingly, the convection pattern disappeared. On the other hand, the lateral gathering of the cells occurred slowly, as could be seen in the space-time diagram (Figure 2A). By increasing the local cell density, gathering at the top could be easily overcome. Hence, after several cycles, a convection pattern appeared again in the localized region. In this time, the wavelength of the pattern became longer than that in constant illumination. This result is expected because of diffusion during long dark period, which decreased the local cell density resulting in long wavelength pattern.

In the case of the shorter period of 20 s, the cells experienced both high and low relative intensity during upward swimming. Although the detailed mechanism is still under investigation, the characteristic behavior observed in the convection patterns may originate from the alternation between upward swimming and random motion. It is known that *Euglena* shows tumbling behavior when the light intensity suddenly increases. Therefore, the upward swimming cells start to swim in a random direction while the light intensity is decreasing, and then tumble in response to a sudden increase in light intensity. On the other hand, downward moving cells experience gradual increase in intensity owing to the shading effect (Vincent and Hill, 1996; Ghorai and Hill, 2005). Notably, some of the cells can respond to strong light and others fail to respond to it and show tumbling. This complicated situation generates a characteristic time evolution of convection pattern, as shown in Figure 2D. Furthermore, the wavelength became slightly shorter than that observed under constant light illumination (Figure 1E). It might originate from failure to deformation of the pattern because of short dark period (5 s), which was shorter than the period to disappearing the pattern (Figure 1C). Uncovering the detailed mechanism of those characteristic observations is a topic for the future works. In addition, with period 8 s, a similar complicated pattern formation was observed on one of the three experiments. Thus, the threshold value of the period was not clear for observing the complicated pattern formation.

Finally, under short-period light, the convection pattern is expected to approach to that observed under constant illumination, in which the convection spots monotonically gathered and formed localized pattern. Because the cells cannot perceive the oscillation but feel an effective constant illumination with a time-averaged intensity under fast oscillation. This is also the future works.

## 5 Conclusion

In this study, we demonstrated one of the simplest setups for self-organized behavior in a temporally changing environment. If the oscillation period was similar in order of time scale for pattern formation behavior, non-trivial characteristic patterns appeared. In our study, the characteristic time was the upward swimming period, which comprised several tens of seconds. Taking a scientific approach to dissect a complex dynamical system behavior, the setup and environmental conditions are typically simplified. As such, it remains important to devise more complicated experimental models to understand these underlying mechanisms. Nevertheless, our work provides a first step toward understanding the spatiotemporal pattern formation of biological systems exposed to periodical environmental fluctuations. Our preliminary experiments clearly showed that a slight change in environmental conditions often induces unexpected and interesting behaviors. We should consider the effect of such a minimally complicated condition as a diorama of more notably complicated natural dynamical systems.

## Data Availability

The original contributions presented in the study are included in the article/Supplementary Material, further inquiries can be directed to the corresponding author.
