# Supplementary material for: Bioconvection pattern of Euglena under periodical illumination
Source: Front Cell Dev Biol. 2023 Mar 17;11:1134002. doi: 10.3389/fcell.2023.1134002 (PMC10063821; doi:10.3389/fcell.2023.1134002)
Supplement: Supplementary file 1 [file DataSheet1.PDF]

Supplementary Material

Supplementary Movie-1:

The convection pattern observed under periodic change in light intensity whose period was 200 s. The lap between each slice is 10 s and the frame rate of the movie is 10 fps. Thus, the speed of movie is 100 times faster. The red period indicates “dark” for *Euglena*, because the cell cannot respond to red light.

Supplementary Movie-2:

The convection pattern observed under periodic change in light intensity whose period was 20 s. The lap between each slice is 20 s and the frame rate of the movie is 10 fps. Thus, the speed of movie is 200 times faster.
